# In Vivo Imaging of Stepwise Vessel Occlusion in Cerebral Photothrombosis of Mice by ^19^F MRI

**DOI:** 10.1371/journal.pone.0028143

**Published:** 2011-12-15

**Authors:** Gesa Weise, Thomas C. Basse-Lüsebrink, Christoph Kleinschnitz, Thomas Kampf, Peter M. Jakob, Guido Stoll

**Affiliations:** 1 Department of Neurology, University of Würzburg, Würzburg, Germany; 2 Department of Physics, EPV, University of Würzburg, Würzburg, Germany; 3 Interdisciplinary Center for Clinical Research Würzburg, University of Würzburg, Würzburg, Germany; University of South Florida, United States of America

## Abstract

**Background:**

^19^F magnetic resonance imaging (MRI) was recently introduced as a promising technique for *in vivo* cell tracking. In the present study we compared ^19^F MRI with iron-enhanced MRI in mice with photothrombosis (PT) at 7 Tesla. PT represents a model of focal cerebral ischemia exhibiting acute vessel occlusion and delayed neuroinflammation.

**Methods/Principal Findings:**

Perfluorocarbons (PFC) or superparamagnetic iron oxide particles (SPIO) were injected intravenously at different time points after photothrombotic infarction. While administration of PFC directly after PT induction led to a strong ^19^F signal throughout the entire lesion, two hours delayed application resulted in a rim-like ^19^F signal at the outer edge of the lesion. These findings closely resembled the distribution of signal loss on T2-weighted MRI seen after SPIO injection reflecting intravascular accumulation of iron particles trapped in vessel thrombi as confirmed histologically. By sequential administration of two chemically shifted PFC compounds 0 and 2 hours after illumination the different spatial distribution of the ^19^F markers (infarct core/rim) could be visualized in the same animal. When PFC were applied at day 6 the fluorine marker was only detected after long acquisition times *ex vivo*. SPIO-enhanced MRI showed slight signal loss *in vivo* which was much more prominent *ex vivo* indicative for neuroinflammation at this late lesion stage.

**Conclusion:**

Our study shows that vessel occlusion can be followed *in vivo* by ^19^F and SPIO-enhanced high-field MRI while *in vivo* imaging of neuroinflammation remains challenging. The timing of contrast agent application was the major determinant of the underlying processes depicted by both imaging techniques. Importantly, sequential application of different PFC compounds allowed depiction of ongoing vessel occlusion from the core to the margin of the ischemic lesions in a single MRI measurement.

## Introduction

Among the non invasive imaging modalities MRI provides high resolution imaging with excellent soft tissue contrast allowing *in vivo* follow up of pathological processes. Commonly, small or ultrasmall superparamagnetic iron oxide (SPIO, USPIO) nanoparticles are used for tracking of labeled cells. Accumulation of these cells in tissues leads to focal signal loss on T2- and T2*-w MRI. However, although relatively low numbers of iron-laden cells can give a strong MRI signal void [1] confounding factors such as blood pool effects and bleedings limit the strength of this technique. Moreover, endogenous iron-laden macrophages can give rise to signal loss even in the absence of contrast agents especially at high field strength [2], [3]. In 2005 ^19^F MRI was introduced as a novel imaging technique for *in vivo* cell tracking after injection of *ex vivo* labeled cells [4]. In contrast to iron contrast agents, ^19^F markers exhibit a unique MRI signal that can be detected directly [5]. Due to the lack of ^19^F background signal in the host's tissue ^19^F MRI is extremely selective for the labeled cells. However, ^19^F MRI requires high numbers of ^19^F spins to accumulate in order to generate sufficient signal-to-noise ratio (SNR). Previous studies have shown that systemic intravenous injection of perfluorocarbons (PFC) leads to significant and spontaneous PFC uptake by cells of the macrophage/monocyte system [6], [7]. By applying *in vivo* ^19^F MRI areas with macrophage infiltration could be visualized in mice in myocardial and cerebral infarctions [7] and in rodent models of acute allograft rejection [8]. We could recently depict neuroinflammation in rats within peripheral nerve lesions by ^19^F MRI [9].

In the present study we applied ^19^F MRI at different stages of cerebral photothrombosis (PT) at high field strength of 7 Tesla (T). In 1985, Watson et al. introduced brain PT as a simple model of focal cerebral ischemia [10]. To achieve thrombosis a photosensitive dye is injected systemically. Subsequent illumination through the intact skull leads to local activation of the dye with free radical formation and photoperoxidation of the endothelium. Endothelial injury is then supposed to mediate clot formation in illuminated vessels. Thus, the pathophysiology of PT lesion development encompasses endothelial damage, breakdown of the blood brain barrier (BBB) and rapid edema formation, but also occlusion of vessels thereby partly mimicking cerebral ischemia [11]. Moreover, PT lesions elicit a strong, but delayed inflammatory reaction [12]. While several factors such as the little ischemic penumbra limit the strength of PT as a model for human stroke [13] it was chosen in this study because lesions show a predictable pattern of macrophage infiltration.

MRI studies at 1.5 T using SPIO in rats showed that in early stages of PT lesion development ongoing vessel occlusion can be visualized by iron-enhanced MRI [14], while delayed application of SPIO depicted neuroinflammation [15]. To elucidate which processes (acute damage versus late neuroinflammation) are visualized in mice with PT we compared lesion development at 7 T after PFC/SPIO injection by ^19^F or T2-w MRI. Our results suggest that ^19^F imaging and iron-enhanced MRI depict vessel occlusion with high sensitivity in the early phase of photothrombotic infarction while at later stages the inflammatory response is covered. The use of two different PFC markers, moreover, allowed detection of stepwise microvascular occlusion in a single PT lesion. In conclusion, the timing of contrast agent application was the major determinant of the underlying processes depicted by these two imaging techniques.

## Materials and Methods

### Photothrombotic infarction

Animal experiments were performed in accordance with institutional guidelines and were approved by the institutional ethics committee for animal welfare of the University of Würzburg, Germany (permit number 62.1-2531.01-23/04; 55.2-2531.01-17/10). Focal cerebral ischemia was induced in 44 adult C57/BL6-mice (25–30 g) by PT of cortical microvessels under inhalation anesthesia with enflurane in a 2∶1 nitrogen/oxygen atmosphere, as described previously [15], [16]. Briefly, a fiber optic bundle of a cold light source was centered stereotactically 2 mm posterior and 2.4 mm lateral from Bregma on the skull exposed via a dorsal midline incision of the skin. 0.2 ml of a sterile-filtered rose Bengal solution were given by intraperitoneal injection and the brain was illuminated for 20 min. Subsequently the skin was sutured and the mice were allowed to recover. This procedure resulted in cone-shaped cortical infarctions. For MRI measurements mice were anesthetized using 1.5% isoflurane in a 2 l/min oxygen atmosphere.

### Contrast agents/markers

#### PFC markers

A perfluoro-15-crown-5-ether emulsion (10% wt/wt, Fluorochem Ltd. Glossop, UK) was used in experiments in which only one PFC emulsion was applied. Production and properties of are described in detail elsewhere [7].

For experiments applying two chemically shifted PFC compounds multi-resonant perfluorooctlybromid (PFC_A_) was used. The nanoemulsion contained 40% wt/wt PFC_A_ (Chempur; Karlsruhe, Germany) and 2.4% wt/wt purified soybean lecithin S75 (Lipoid; Ludwigshafen, Germany) in an isotonic buffer (7 mM Na_2_HPO_4_ - 2 H_2_O, 3 mM NaH_2_PO_4_ - 2 H_2_O, 2.5% glycerol, pH 7.4) and was prepared as previously reported [7]. High pressure homogenization (75 MPa, 10 cycles) with Emulsiflex C5 (Avestin; Mannheim, Germany) resulted in a particle size of ≈240 nm as determined by dynamic light scattering (average of at least ten runs measured at the stationary level) using Zetatrac™ (Particle Metrix; Meerbusch, Germany). The nanoemulsion was sterilized by autoclavation and stored at 6°C until application. Besides PFC_A_ the single-resonant perfluoro-15-crown-5-ether (PFC_B_) was used. The 30% v/v PFC_B_ emulsion was obtained from Celsense Inc. (Pittsburgh, PA, USA).

#### SPIO contrast agents

SPIO particles (Resovist™, Bayer Schering Pharma AG, Berlin, Germany) were used in a dosage of 0.2 mmol Fe/kg body weight as described in previous studies at 1.5 T [15], [17].

### Hardware

All measurements were performed on a 7 T Bruker Biospec System (Bruker BioSpin GmbH, Reinstetten, Germany) at room temperature. For *in vivo* ^1^H and ^19^F imaging a home-built surface coil adjustable to both frequencies was used. *Ex vivo* imaging of the fixed mouse brains was performed with a home-built solenoid coil. Additionally, a home-built, actively decoupled ^19^F birdcage coil in combination with an actively decoupled ^19^F receive-only surface coil was used for the experiments with two PFC compounds. Even though the solenoid and the birdcage with integrated surface coil were optimized for the ^19^F resonance frequency, their performance was still sufficient to acquire ^1^H anatomical background images.

### *In vivo* ^1^H/^19^F imaging

#### Experiments in the acute phase of lesion development

In order to visualize and quantify different stages of acute “ischemic” damage PFC emulsion was applied via the tail vein in a dosage of 250 µl either directly after the end of illumination (n = 8) or two hours later (n = 8). Two mice with cortical infarctions that obtained 250 µl of 0.9% sodium chloride intravenously instead of PFC served as negative controls.

MRI scans were performed 24 hours after PFC injection. In n = 8 animals (0 h/2 h = 4/4) 2D single slice experiments were performed. In all experiments the slice was located axially through the middle of the infarction using information obtained from scout scans. For ^1^H reference images, a turbo spin echo (TSE) sequence was used (echo time (TE)/repetition time (TR): 40 ms/5000 ms; inter-echo time: 10 ms; turbo factor: 8; field-of-view (FOV): 25×25 mm; matrix: 256×256; slice thickness (SI): 2 mm; number of averages (NA): 1). Regarding ^19^F imaging 2D steady-state free precession CSI (SSFP-CSI) sequences [18] were performed with the same geometry as the TSE scans (pulse shape: hermite; pulse bandwidth: 5400 Hz; acquisition time(T_AQ_)/TR: 10.3 ms/13.6 ms; FOV: 25×25 mm; spectral points: 512; matrix: 41×41; SI: 2 mm; NA: 158). The overall protocol time was <1.5 hours.

To be able to quantify the lesion volume 3D experiments were performed in additional n = 8 mice (0 h/2 h = 4/4). Thus, 3D acquisition weighted ^19^F SSFP-CSI data were acquired [19]. The FOV was set to 25×25×20 mm, the matrix and the NA were adjusted to allow the same nominal spatial resolution and total imaging time as a 40×40×20 and 8 times averaged scan with full coverage of the k-space. To eliminate banding artifacts an additional 3D ^19^F SSFP-CSI scan with a 180° phase shift alternation of the excitation pulse was performed. Additional multislice (SI = 1 mm) ^1^H TSE reference scans were performed (otherwise same parameters as described above). For the quantification study the overall protocol time was ≈2.5 hours.

#### Lesion maturation experiments

In order to follow the ^19^F signal during lesion maturation qualitatively n = 2 (2 h) mice underwent additional 2D single slice MRI scans 3, 8 and 10 days after PT as described above.

#### Different stages of acute “ischemic” damage visualized in a single experiment

To visualize ongoing vessel occlusion in the acute “ischemic” stage n = 3 mice received 125 µl PFC_A_ emulsion intravenously directly after the end of illumination. Two hours later 125 µl of PFC_B_ was applied into the same animals.

^1^H and ^19^F scans were performed at days three and eight after PT. Multislice ^1^H TSE datasets of the mouse brain were acquired for anatomical references (slices: 28; SI: 1 mm; FOV: 25×25 mm; matrix: 256×256). 3D ^19^F SSFP-CSI datasets without slice selection were acquired using the same geometry as the ^1^H TSE datasets (pulse shape: hard pulse; pulse bandwidth: 50000 Hz; FOV: 25×25×28 mm; matrix: 41×41×14; NA: 6). The pulse bandwidth was increased to 50000 Hz to excite the complete spectrum of the PFC_A_ compound. Otherwise the same parameters were used as described above. The protocol time was <1 hour.

#### Neuroinflammation experiments in the late stage of PT

In n = 8 mice the PFC emulsion was administered at day 6 when secondary invasion of hematogenous macrophages is supposed to happen [12]. MRI measurement was carried 48 hours later at day 8 to allow labeled macrophages more time to invade into the photothrombotic lesion. The same scans were performed as described for the volume quantification experiments. The protocol time was <2.5 hours.

### *Ex vivo* ^1^H/^19^F imaging

*In vivo* MRI of mice injected at day 6 did not exhibit fluorine signal within the lesion. To clarify whether a lack of signal led to insufficient sensitivity of *in vivo* imaging post mortem scans of the isolated brain were performed. Mice were sacrificed after the *in vivo* MRI measurement at day 8 by perfusion with 0.9% sodium chloride followed by 4% paraformaldehyde in deep anesthesia. The brains were removed in toto and fixed overnight in 4% paraformaldehyde.

For ^1^H reference images, a multislice TSE sequence was used (FOV: 15×15 mm; matrix: 128×128; SI: 1 mm; 36 slices, otherwise same parameters as *in vivo* ^1^H/^19^F imaging). Regarding ^19^F imaging 3D acquisition weighted SSFP-CSI scans were acquired with the same geometry as the TSE scans. The matrix and the number of averages were adjusted to allow the same nominal spatial resolution and total imaging time as a 30×30×18 and 256 times averaged scan with full coverage of the k-space. Otherwise same imaging parameters as described in the *in vivo* ^1^H/^19^F imaging section were applied. The overall *ex vivo* protocol time was 16 hours.

### *In vivo* SPIO-enhanced MRI

In accordance with the PFC injection protocol a parallel group of mice (n = 15) received SPIO intravenously immediately after the end of illumination (n = 4), two hours later (n = 4) or at day 6 (n = 7).

MRI measurements were performed 24 hours after systemic administration of the contrast agent. For MRI T2-w imaging the same single slice TSE sequences as for the anatomical reference in the ^19^F *in vivo* experiments were used. Furthermore, multislice (SI = 1 mm) TSE measurements were performed. The total protocol time for the *in vivo* SPIO measurements was <30 minutes.

### *Ex vivo* SPIO-enhanced MRI

Mice with delayed application of SPIO at day 6 were sacrificed after the *in vivo* MRI scan by perfusion with 0.9% sodium chloride followed by 4% paraformaldehyde in deep anesthesia. The brains were removed in toto and fixed overnight in 4% paraformaldehyde. Two post mortem T2*-w 3D fast low angle shot (FLASH) scans of the brain were obtained (TE/TR: (4 ms/20 ms)/50 ms; FOV: 15×15×36 mm; matrix: 150×150×360; NA: 4). Each *ex vivo* FLASH scan lasted three hours.

### Post Processing

All post processing was done in MATLAB (The MathWorks Inc., Natick, USA). Thereby semi-automated, home-written algorithms were used for quantification experiments as described below. Quantification was performed independently by three different persons.

#### ^19^F imaging

To generate ^19^F images only the signal of the ^19^F peak was integrated. Furthermore, a threshold was applied to the ^19^F data and afterwards overlaid with the ^1^H TSE data. For each overlay image the threshold was adjusted individually.

Regarding quantification, sum-of-squares (SOS) reconstructions were generated of the data with and without alternating phase to minimize banding artifacts in a first step. In a second step the signal of the ^19^F peak was integrated to generate ^19^F images. Afterwards the 3D ^19^F dataset was zerofilled to the size of the ^1^H reference dataset (256×256×20). For all ^19^F datasets, the volume of the infarct containing ^19^F signal was evaluated by only counting pixels with ^19^F signal greater than a threshold value set by the investigator. Thereby, only the pixels of the ^19^F dataset in the infarct area were regarded. The infarct area was manually segmented by the investigator using the contrast of the ^1^H reference dataset (positive T_2_ contrast) prior to the ^19^F quantification.

#### SPIO-enhanced MRI

For quantification, the infarct area was manually segmented by the investigator using the contrast of the ^1^H reference dataset (positive/negative T_2_ contrast). In a second step the volume of the infarct showing signal voids was calculated by only counting pixels with SNR lower than a threshold value defined by the investigator.

### Histology

After the *ex vivo* MRI PFA-fixed brains were washed in phosphate-buffered saline (PBS) and embedded in paraffin. Subsequently 5 µm thick coronal sections were cut at multiple levels through the infarction, deparaffinized with xylene, rehydrated and washed in water and PBS. For iron detection tissue sections were rinsed in deionized water and immersed in Perl's solution containing 2% potassium ferrocyanide and 2% HCl at a 1∶1 concentration for 30 minutes. Sections were then rinsed in deionized water and either dehydrated, counter stained with hematoxylin-eosin and coverslipped or further processed for immunohistochemistry. Immunohistochemistry was performed using von Willebrand Factor (vWF) (polyclonal rabbit anti-mouse IgG, ab6994, 1∶800; Abcam, USA), an endothelial cell marker, as primary antibody upon the same sections. Enzymatic antigen retrieval was performed with 0.1% pronase for 10 min at 35°C. Binding of the antibodies to cells was visualized by a biotinylated goat anti-rabbit IgG secondary antibody (1∶100, Vector laboratories, USA). For negative controls, the primary antibody was omitted from the diluent.

## Results

### ^19^F and iron-enhanced MRI in early stages of infarct development

In a first set of experiments mice received either PFC emulsion or SPIO directly after the end of illumination of the brain or with two hours delay. Control animals with cortical infarctions but no PFC injections did not exhibit fluorine signal under inhalation anesthesia with isoflurane (data not shown).

**Figure 1:**
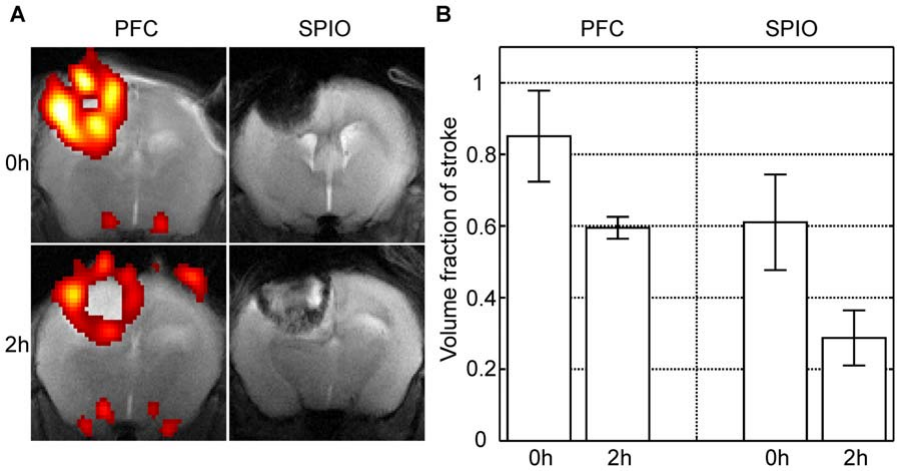
*In vivo* imaging patterns of ^19^F and SPIO-enhanced MRI in the acute stage of cerebral photothrombosis. (A) All images were acquired 24 hours after PT induction. The ^1^H/^19^F overlay shows fluorine signal within the entire cortical infarction when PFC were applied directly after the end of illumination (left, upper image). Delayed injection two hours later led to a rim-like signal in the boundary zone (left, lower image). Note the similar distribution pattern of signal loss on T2-w MRI when SPIO were administered immediately after illumination (right, upper image) and with two hours delay (right, lower image). The relative volume of iron-induced signal loss and ^19^F signal in proportion to the total infarct volume decreased from 0.85±0.13/0.61±0.13 to 0.59±0.03/0.29±0.08 (PFC/SPIO) within the first two hours after illumination (B) (both cases p<0.05). When corresponding time points of SPIO/^19^F marker injection were compared a significant difference was found between the volume fraction showing ^19^F signal and the signal voids in SPIO-enhanced MRI (p<0.05).

**Figure 2:**
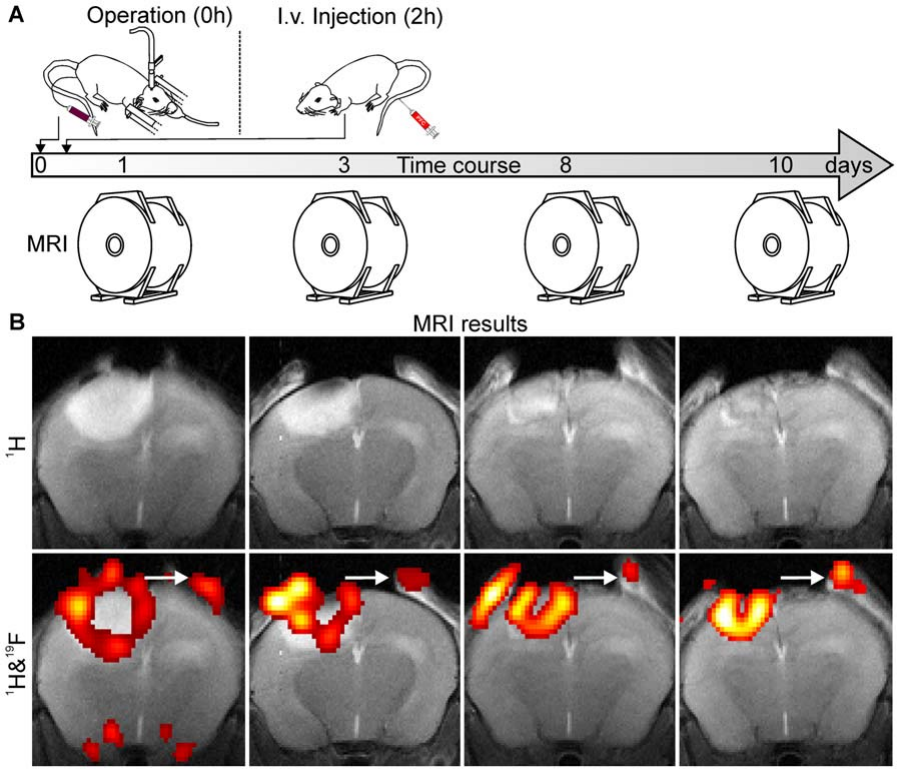
Development of the ^19^F signal during lesion maturation. Individual animals that received PFC two hours after photothrombotic infarction were scanned sequentially on days 1, 3, 8 and 10 (A). On ^1^H-TSE lesions initially appeared hyperintense due to increased T_2_ values caused by tissue damage and edema formation. Over time proton signal changes decrease leading to an almost isointense tissue signal at day 8 (B). The rim-like fluorine signal seen one day after PT induction persisted until day 10 and appeared even more intense with shrinkage of the lesion over time. An additional fluorine signal could be observed at the site of skin incision (arrows).

Administration of PFC directly after illumination led to a bright ^19^F signal throughout the entire cortical infarction as shown on the *in vivo* MRI scan 24 hours later (Fig. 1A). When PFC were injected with two hours delay lesions displayed a ring-like fluorine signal at the lesion margins (Fig. 1A). The rim-like fluorine signal seen at day 1 persisted when individual 2 h-animals were scanned again during further lesion maturation at days 3, 8 and 10 (Fig. 2). On ^1^H-TSE images the lesions initially appeared hyperintense. Within the first week after PT induction signal changes decreased leading to an almost isointense tissue signal at day 8 (Fig. 2B). The semilunar ^19^F signal appeared more intense with shrinkage of the lesion over time (Fig. 2B).

The distribution of the signal loss on T2-w MRI after SPIO injection closely resembled the ^19^F signal following PFC administration directly after illumination or with a delay of 2 hours. Whereas in animals which obtained SPIO immediately after completion of PT markedly hypointense cortical lesions were depicted on T2-w MRI, injection with 2 hours delay led to a hyperintense infarct core with a hypointense ring at the lesion margins (Fig. 1A). Furthermore, using statistical t-tests the quantification of each of the three independent researchers revealed a significant difference between the ^19^F volume fractions of the lesions when the PFC emulsion was injected 0 h or 2 h after the illumination (p<0.05). Similarly, the volume fractions showing signal voids in SPIO-enhanced MRI 0 h and 2 h after the illumination significantly differed (p<0.05). However, when corresponding time points of SPIO/PFC injection were compared a significant difference was found between the volume fraction showing ^19^F signal and the signal voids in SPIO-enhanced MRI (both cases: p<0.05). Thus, the mean volume fraction and mean error of the lesion exhibiting ^19^F signal was 0.85±0.13/0.59±0.03 (0 h/2 h) and 0.61±0.13/0.29±0.08 (0 h/2 h) for the iron-enhanced experiments (Fig. 1B). A relative measurement of the volume fraction was chosen due to a certain interindividual variance of the stroke volumes. Therefore, total quantification could have generated misleading results with high standard deviations.

#### In vivo visualization of different stages of acute “ischemic” damage

**Figure 3:**
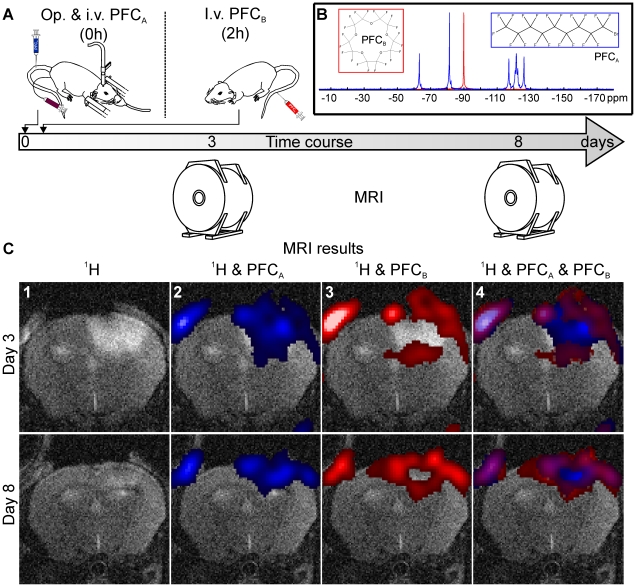
*In vivo* visualization of ongoing vessel occlusion. A subgroup of animals received two chemically shifted PFC compounds sequentially. PFC_A_ with a multi-resonant perfluorooctlybromid was administered immediately after the end of illumination while single-resonant PFC_B_ with a peak at 90 ppm was injected with two hours delay (A,B). Representative coronal slides of the mouse brain 3 and 8 days after induction of the photothrombotic infarction are shown (C). Administration of the first emulsion (PFC_A_, blue) directly after induction of PT led to a fluorine signal throughout the cortical infarction (C2) while the second ^19^F marker (PFC_B_, red) that was applied two hours later accumulated at the outer margins sparing the center of the infarcted zone (C3). Combined overlay of the ^1^H TSE data with data from both PFC compounds allowed visualization of the distinct marker distribution in a single 3D *in vivo* measurement (C4). Similar to the previous experiments an additional ^19^F signal of both compounds can be observed at the site of the skin incision.

Additional 3D CSI experiments with two chemically shifted PFC compounds were performed which allowed differentiation of both markers and thus visualization of ongoing vessel occlusion in a single MRI measurement (Fig. 3). Administration of the first emulsion (PFC_A_) directly after induction of PT led to a fluorine signal throughout the cortical infarction (Fig. 3C). The second ^19^F marker (PFC_B_) that was applied two hours later, however, accumulated at the outer margins sparing the center of the infarcted zone (Fig. 3C). By merging the signals on a ^1^H background image the different spatial distribution of the compounds could be identified in a single measurement (Fig. 3C).

### ^19^F and iron-enhanced MRI in late stages of infarct development: Visualization of neuroinflammation

**Figure 4:**
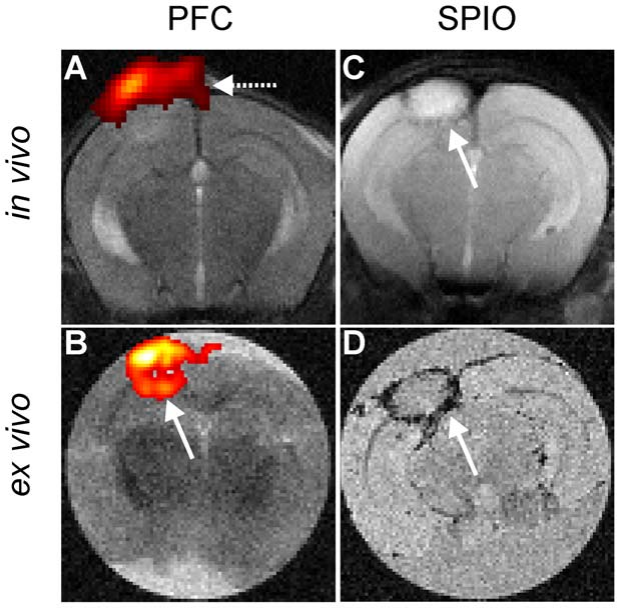
*In vivo* and *ex vivo* ^19^F and SPIO-enhanced MRI in late stages of infarct development. (A) shows an *in vivo* ^1^H/^19^F overlay of the infarcted zone when PFC were administered six days after PT induction. Note the lack of fluorine signal within the lesion. The ^19^F marker is only visible at the site of skin incision (dashed arrow). Post mortem scans of the removed brain with long acquisition times revealed a fluorine signal in the ischemic lesion (arrow in panel B). Application of SPIO led to a ring-like signal loss indicative of macrophage infiltration (arrows in panel C/D) which was weakly visible on *in vivo* T2-w MRI at 7T (C), but very prominent on *ex vivo* T2*-w MRI scans (D).

When the PFC emulsion was administered with a delay of 6 days after PT and animals were scanned at day 8 no fluorine signal was detectable within the lesions on *in vivo* ^19^F MRI scans (Fig. 4A). The ^19^F marker was only visible at the site of the skin incision on the skull (Fig. 4A, arrow). However, when mice were sacrificed after the *in vivo* measurement and post mortem scans of the isolated brain with long acquisition times and the use of a more sensitive solenoid coil were performed a fluorine signal in the ischemic lesion was regularly detectable (Fig. 4B). After SPIO application at day 6 a small rim of signal loss was regularly visible on *in vivo* T2-w MRI (Fig. 4C) as shown previously for rats at 1.5 T [15]. The ring-like hypointensity was even more prominent on *ex vivo* T2*-w MRI scans of the removed brain (Fig. 4D). Proton signal changes in PT decrease during the first week leading to an almost isointense infarct signal at day 8 so that quantification of the ^19^F signal in relation to the total infarct volume was impossible at this late stage of lesion development.

### Histological analysis

**Figure 5:**
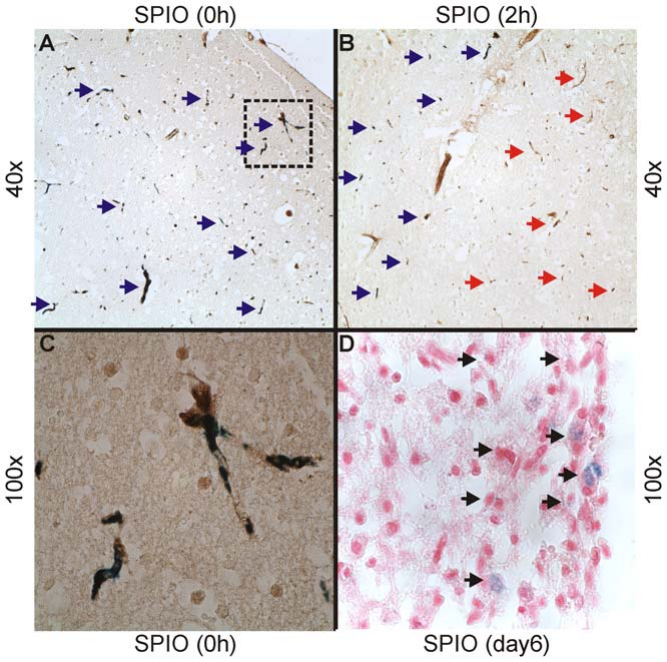
Histological analysis of the infarctions at different time points after photothrombotic stroke. Localization of SPIO particles in focal cerebral infarctions 24 hours after PT induction. In (A) SPIO particles were injected directly after the end of illumination, in (B) with a delay of two hours. Coronal paraffin sections cut through the infarction were additionally immunolabeled with vWF antibody to unequivocally identify cerebral endothelial cells. SPIO particles by Perl's stain appear in blue. Note iron deposits throughout the core of the infarcted area in cerebral vessels stained by vWF in (A). (B) shows the outer border of the ischemic zone on the left lower part and the core region on the right upper part. The iron entrapment after two hours delayed injection is now restricted to vessels in the left border zone (blue arrows) while vWF positive vessels in the center of the lesion are devoid of iron particles (red arrows). A higher magnification illustrates the localization of the iron particles inside the vessels identified by staining against vWF. (D) In contrast, blue appearing iron particles are no longer found in the vessel lumina but are located within the cytoplasm of cells (black arrows) representing macrophages [14], [22] when SPIO are applied at late stages of lesion development (day 6). Magnifications (A) 40×, (B) 40×, (C) 100×, (D) 100×.

Macroscopically the circular PT lesions could be identified at the frontoparietal cortex as shown previously [20]. Prussian blue staining on coronal sections cut through the infarction of mice sacrificed one day after PT induction revealed entrapment of iron particles throughout the infarct core when SPIO were applied directly after the end of the illumination (Fig. 5A). The intravascular location of the iron deposits was confirmed by co-staining of identical sections with antibodies against vWF, an endothelial cell marker (Fig. 5A–C). Iron particles could only be detected within the vessel lumina in the infarct region, but not in the brain parenchyma or remote cortex. After two hours delay the iron nanoparticles were trapped in the cortical vessels at the outer margin of the infarction while vessel thrombi in the center of the lesion were devoid of iron particles (Fig. 5B). This finding supports our MRI results depicting ongoing vessel occlusion despite cessation of the illumination exclusively in the periphery of the photothrombotic lesion. At late lesion stages eight days after PT induction accumulation of blue iron particles was no longer found in the vessel lumina but intracellular within the infarcted zone (Fig. 5D). These findings correspond to previous results obtained in rats [14].

## Discussion

As principal finding we show that ^19^F MRI can visualize ongoing vessel occlusion and cellular infiltration in cortical PT, a model of focal cerebral ischemia. The timing of contrast agent application was the major determinant of the underlying processes depicted by ^19^F MRI.

### ^19^F and iron-enhanced MRI in early stages of infarct development

When the PFC emulsion was injected directly after PT induction the fluorine signal was displayed throughout the entire lesion. Two hours delay led to a fluorine rim at the periphery of the infarction. Both changes were present during the acute phase of lesion formation in which hematogenous macrophages are not yet significantly involved [12], [15]. Signal alterations visualized by ^19^F imaging during acute PT closely resembled the pattern of signal loss on T2-w MRI after application of SPIO. However, the relative volume fractions showing a ^19^F signal or signal voids in SPIO-enhanced MRI at corresponding injection time points differed significantly. This might on one hand be due to partial volume effects leading to an overestimation of the ^19^F signal as only a relatively low spatial in-plane resolution could be obtained. On the other hand, PFC emulsions and SPIO have different blood clearance times. The PFC emulsion which was used in the present study for quantification experiments was shown to circulate in the blood up to two days [7]. In contrast, SPIO concentrations significantly decline in the circulation within the first hour after application due to uptake of the iron particles in the reticuloendothelial system of the liver [21]. On tissue sections iron deposits could be exclusively found in vessels of the infarct core when SPIO were applied directly after the end of the illumination while SPIO injection two hours later lead to intravascular entrapment of the iron particles mainly at the outer margins of the lesion indicating ongoing vessel occlusion despite cessation of the illumination. The same mechanism very likely underlies the fluorine signal observed in the early phase of photothrombotic stroke in our study.

### ^19^F and iron-enhanced MRI in late stages of infarct development

Unlike the *in vivo* ^19^F MRI in the early stage of infarct development no intracranial fluorine signal could be detected *in vivo* when the PFC emulsion was applied with a delay of six days after PT. However, *ex vivo* ^19^F MRI with long acquisition times and use of a more sensitive coil disclosed a fluorine signal within the PT lesions at this late lesion stage most likely reflecting the infiltration of fluorine-labeled macrophages from the blood.

Several groups have already shown the uptake of PFC in activated macrophages [6], [7], [8]. In their work, Flögel and colleagues [7] focused on cellular infiltration after myocardial infarction, but also studied ^19^F MRI in the PT model. In contrast to our work a fixed scheme with two injections of the PFC emulsion, two hours after PT induction and six days later, was used. Interestingly, the two hours delayed PFC administration in our study led to a ring-like appearance similar to the ^19^F TSE MRI scans shown after day 7 by Flögel et al. [7]. Their histological analysis performed seven days after PT induction confirmed co-localization of rhodamine-labeled PFC with CD11b-positive macrophages/microglia in the PT lesions. Importantly, the rhodamine-labeled PFC emulsion was only used for the second injection at day 6 indicating that the observed macrophage/microglia labeling could only be due to this injection. Although in our study no intracranial ^19^F signal was detectable *in vivo* when PFC were applied at day 6 and the first injection two hours after illumination was omitted, *ex vivo* scans of the removed brain after long acquisition times regularly revealed the fluorine marker within the infarcted zone. Thus, in our hands the fluorine signal generated by intravascular trapping of the PFC emulsion early during PT was much stronger than the signal intensity obtained by macrophage invasion into the mature PT lesion at later points of time.

Previously it was shown in rats that late SPIO application at days 5 or 6 after PT leads to a hypointense rim around the lesion on *in vivo* 1.5 T MRI due to accumulation of SPIO-laden hematogenous macrophages [15], [22]. In our 7 T study in mice this finding could be confirmed by *in vivo* T2-w MRI at day 8 showing a small rim of signal loss at the boundaries of the lesions. T2-w instead of T2*-w MRI was chosen since T2*-w in vivo studies at this high field strength can be hampered by significant susceptibility artifacts. Importantly, histological evaluation at this late stage of lesion development showed iron deposits merely intracellular, but not in the vessel lumina.

It is well established that PT lesions induce early and persistent breakdown of the BBB within the lesion and transiently for 24 h within the entire, morphologically intact ipsilateral cortex [23], [24]. Although PFC and SPIO are likely to pass through a broken BBB, the timely and locally restricted patterns of ^19^F (this study) and SPIO enhancement [14], [15] are not compatible with passive accumulation of these contrast agents within the lesion as seen with the conventional MR contrast agent Gadolinium (Gd)-DTPA [15]. PT lesions become Gd-DTPA enhanced on T1-w MRI within hours after induction and Gd-DTPA-uptake lasts up to weeks [25]. In contrast ^19^F- and SPIO-enhancement follows distinct patterns reflecting either acute vessel occlusion or inflammation, but not merely leakage of the BBB. The lack of perivascular or parenchymal iron accumulation in the acute PT lesions also argues against passive diffusion of the contrast agents into the brain parenchyma.

^19^F MRI is a relatively new experimental imaging technique which still depends on customized coil manufacture and individual sequence adaption. This could explain some of the discrepancies described above. Since the fluorine signal in the PT model is located near the surface, we used self-made surface coils for the *in vivo* experiments. This setup yields improved SNR compared to a volume resonator [26]. Moreover, a ^19^F SSFP-CSI sequence instead of a ^19^F TSE MRI sequence, a reduced in-plane resolution and a longer acquisition time were chosen which could explain the higher sensitivity of our approach during early PT. However, sensitivity of our set-up appears not to be strong enough for *in vivo* detection of intracerebral cell-bound fluorine while it worked well in sciatic nerves [9]. Improved hardware and adapted ^19^F sequences might help to overcome this restriction for imaging of brain neuroinflammation in the future.

The key advantage of ^19^F MRI is its high selectivity due to the negligible ^19^F background signal in mammals. With use of iron-enhanced MRI it often remains difficult to unequivocally assign alterations in local contrast to accumulation of iron nanoparticles and not to other field inhomogeneities. Moreover, the prominent iron-related signal loss eight days after PT in our study could partly be caused by intrinsic iron loading of macrophages during vascular degeneration [3] or following hemorrhage as shown by Jolkkonen et al. [2]. Thus, ^19^F MRI might help to overcome the limitations of iron-enhanced MRI in the future (reviewed in [27]). Another virtue of ^19^F MRI is that it allows *in vivo* discrimination of different PFC compounds with large chemical shifts in acceptable measurement times [28]. Thus, using a photothrombotic lesion model we were the first to monitor stepwise thrombus formation from the core of the infarction to its outer margin in a single MRI measurement. Further studies are required to clarify whether ^19^F MRI can be applied to stroke models with different lesion dynamics.

Taken together we have shown that the application scheme of PFC has a major impact on the interpretation of MR findings in different organs. In brain ischemia vascular trapping during thrombus formation is an important component which may prevail over macrophage infiltration. Overall, it seems that visualization of cellular infiltration by ^19^F imaging in the central nervous system is much more difficult than in other organs such as the heart, lung and peripheral nerve [6], [7], [9] due to lower signal intensities and requires further technical development.
